# Political Populism, Institutional Distrust and Vaccination Uptake: A Mediation Analysis

**DOI:** 10.3390/ijerph19063265

**Published:** 2022-03-10

**Authors:** Almudena Recio-Román, Manuel Recio-Menéndez, María Victoria Román-González

**Affiliations:** Department of Economics and Business, University of Almería, Carretera de Sacramento s/n, 04120 Almería, Spain; mrecio@ual.es

**Keywords:** vaccine hesitancy, populism, consumer behavior, social marketing

## Abstract

Politics is ubiquitous in public health, but vaccines had never been weaponized to instill distrust to gain political advantage. In pandemic and post-pandemic scenarios, populist political parties could use vaccine-related issues to generate distrust in evidence-based knowledge. Therefore, some questions arise. What impact could populist political parties impinge on vaccination uptake rates through sowing political discontent? What could the medical institutions do to avoid the adverse effects that these political strategies could infringe? For answering these research questions, we first hypothesized that vaccine uptake was negatively associated with distrust in the institutions. Furthermore, we analyzed whether populism mediates this relationship. In doing so, we hypothesized a positive association between distrust and populism, because populists, mainly fueled by politically discontent citizens, offer hope of a better future, blaming their misfortune on the actions of the elite. Additionally, we hypothesized that those citizens with a higher level of political dissatisfaction, following the claims of the populist political parties, will have lower vaccine uptake results, because they will be discouraged from making the efforts to counter the pandemic. Based on a survey carried out by the European Commission that covered 27 E.U. + U.K. countries (totaling 27,524 respondents), this paper proves that an individual’s political discontent fully mediates the relationship between distrust in institutions and vaccine uptake. Targeting the vaccine-hesitant population is quite convenient for populists because they only need to convince a minority of citizens not to be vaccinated to achieve their destabilizing goals. New outbreaks will appear if the minimum herd immunity coverage is not reached, reinforcing a vicious circle of distrust in elites, in consequence. For tackling this matter, recommendations are given to institutional managers from a social marketing standpoint.

## 1. Introduction

COVID-19 has brought forward a new role for politics, in the global vaccines scenario [1]. Governments buy vaccines and decide who will be jabbed, and when. Traditionally, the pharmaceutical industry and the health system’s institutions (at national, regional, or local levels) took these decisions. In addition, politics played, and still plays nowadays, an essential role in several other vaccine-related dimensions: research and development, procurement, production, and marketing activities [2]. Hence, politics is ubiquitous in public health [3], but vaccines have never been weaponized to instill distrust to gain a political advantage [4].

An upward trend supporting populist parties is present in developed and developing countries, implying a significant challenge for universal healthcare [5]. Several studies showed that votes for populist parties are mainly based on dissatisfaction with the political establishment [6]. Political populism defends ordinary people in contrast with the real or perceived elite. The elite usually refers to mainstream political parties, the media, the upper classes, intellectuals, and, in the territorial scope of this work, the European Union [7]. When applied to healthcare issues, medical populism [8] is based on a distrust of evidence-based policy interventions and the condemnation of technocratic knowledge [3,9,10]. Worsening population health may cause more significant social discontent and the growth of populist sentiments [11]. Hence, populist leaders will exploit health crises for political gain [7,11].

Resistance to vaccination could be seen in disenchanted citizens as a way to express their discontent [12]. The populist approach appeals to groups of citizens that have been left behind for various reasons, including the consequences of the globalization process [3,13]. Populist leaders take advantage of their discontent, blaming their misfortune on the elite’s actions. Populist politicians offer politically dissatisfied people the hope of a better future, attracting their votes [13,14]. Consequently, a worrisome circle occurs: populism fuels the spread of infectious diseases and infectious diseases fuel populism [13]. Several real examples illustrate that medical populism is not occasional but a frequent response to pandemic emergencies [8]. A common medical populism framework for action was shared in cases, such as the H.I.V. denialism in South Africa [15], the measles-rubella vaccine scare in Ukraine [16], the Nigerian boycott of the polio vaccine [17], the Dengue vaccine scandal in the Philippines and other countries of the global south [18], the West African Ebola virus epidemic scare [19], the anti-vaccination movement in Italy [20], and the more recent responses to the COVID-19 pandemic [13], in the U.S.A., Brazil, the Philippines, Poland, Russia, India, and the United Kingdom. Populist governments implemented soft policy measures and discouraged citizens’ efforts to counter the pandemic in all these cases. As a result, populist-governed countries are hit worse by a pandemic than conventional ones [21].

The main goal of this paper is to measure how the relationship between distrust of institutions and vaccine uptake is mediated by political dissatisfaction. To the best of our knowledge, no one has ever studied it before.

## 2. Conceptual Background

### 2.1. Trust and Vaccine Uptake

Public trust in vaccination has declined in the last two decades [22]. Following Larson [22], trust is the “relationship that exists between individuals, as well as between individuals and a system, in which one party accepts a vulnerable position, assuming the best interests and competence of the other, in exchange for a reduction in decision complexity”. Vaccine-related information and decision-making are technically complex enough to rely on people’s confidence in the different health system components [23]. There are several levels of trust involved in the vaccine uptake process. The core of the vaccine’s trust framework comprises three items [24]: the trust in the vaccine, the provider, and the political system. Quantitative studies that examined the relationship between trust in the health system and vaccine uptake found a positive association among them [25,26,27,28]. However, other studies showed that the variation in trust between vaccines and healthcare providers does not explain the variation in vaccination coverage [29,30]. Therefore, to study the variability in the influence of trust in vaccine uptake, we must mainly consider other social institutions that could influence the vaccination decision-making process. Among these social institutions, we thought it essential to analyze the information media, the political parties, the regional or local public authorities, the national government, the national Parliament, and, due to the territorial scope of this paper, the European Union [24,31]. These could explain how vaccine hesitancy increases worldwide, while having highly effective vaccines [32]. Building on Recio-Román et al. [32], we measured trust using a simplified scale that considers the social institutions mentioned earlier (from now on referred to as Distrust because it was reverse coded). Following the previous reasoning, we expected that the higher a person’s distrust in the institutions, the lower the vaccine uptake will be.

**Hypothesis** **1** **(H1).**  *The higher the Distrust in the institutions, the lower the Vaccine Uptake is*.

### 2.2. Political Dissatisfaction, Populism, Vaccine Hesitancy, and Vaccine Uptake

Vaccines have a differential characteristic from other medicines; to be effective, it is necessary to immunize a high percentage of the population. Also known as herd immunity—the level at which immunization coverage must be maintained to be effective—this percentage varies between 80% and 90% in most vaccines [23,33]. If this level is not reached and maintained, the risk of new outbreaks increases—also called the small pockets issue. Vaccine reluctance and refusal are not the same things. Vaccine hesitancy—the reluctance or refusal to vaccinate despite the availability of vaccines—lies between those who accept all vaccines without any doubt and those who reject all vaccines without any doubt [34]. It is an increasing trend all around the world [35].

The existing body of literature on the rising support of populist movements across the globe emphasizes the highly significant role of political discontent [36,37,38]. Therefore, political dissatisfaction is critical in developing definitions of populism [8,39,40]. Populism is a consequence of democratic dysfunction, caused by the feeling that democratic institutions are not working well [41,42]. Following Kitschelt [43], populism is an expression of dissatisfaction with existing modes of organized elite mass political intermediation and the desire to abandon the intermediaries that stand between citizens and rulers. Furthermore, political discontent increases the likelihood of stable voting for populist parties [44]. Vaccine hesitancy matches flawlessly in populist agendas. Both share the profound distrust in elites and experts as their main drivers [45]. High levels of distrust and polarization are fertile areas for strengthening dissatisfaction against the elite [46].

**Hypothesis** **2** **(H2).**  *Distrust in institutions is positively associated with political discontent (populism)*.

Political ideology influences how policymakers address and solve healthcare issues [47]. Populist political parties know that vaccines are fertile ground for instilling doubt and gaining from polarized debates [48]. In pandemic and, mainly, post-pandemic scenarios, populists polarize pros and cons on vaccines, linking them with any other factionalizing feelings (anti-chemical, anti-science, anti-migration, anti-abortion, anti-government, etc.) [49]. Targeting the vaccine-hesitant population is very convenient for populists because they only need to convince a minority of citizens not to be vaccinated to achieve their destabilizing goals. New outbreaks will appear if the minimum herd immunity coverage is not reached, reinforcing a vicious circle of distrust in elites [23,33].

**Hypothesis** **3** **(H3).**  *Political discontent (populism) is negatively associated with vaccine uptake*.

**Figure 1:**
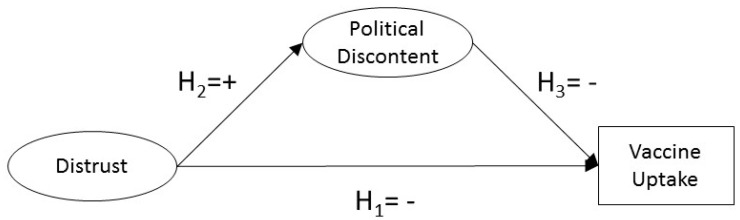
Conceptual model and hypotheses.

Hence, in this paper, considering political discontent as a proxy for political populism, we study how Populism mediates between Distrust and Vaccine Uptake. We expect that the higher the Distrust, the lower the Vaccine Uptake—total effect. As Figure 1 depicts, we hypothesized that those with a higher level of political dissatisfaction have lower vaccine uptake results—indirect effect. The total effect not explained by the populism mediation comprised the direct effect.

Finally, several studies also consider sociodemographic variables as moderators of vaccine uptake [12,50,51,52]. However, it is remarkable that they reach inconclusive or contradictory findings [53]. These could be explained by considering them as potential confounders of factors that determine vaccine uptake. Even though they could be related to vaccine uptake, they cannot explain its variation. They could help to target purposes, but the design of the planned intervention must rely on the other drivers that the model offers.

## 3. Materials and Methods

### 3.1. Sample

The data stemmed from the EUROBAROMETER survey 91.2 carried out between 15 and 29 March 2019 by the company Kantar Public, at the request of the European Commission [54]. The dataset was accessed through GESIS (Leibniz-Institute für Sozialwissenschaften, University of Cologne, Germany) at https://www.gesis.org/ (accessed on 16 October 2021). The EUROBAROMETER is part of wave 91.2 and covers the population of the respective nationalities of the European Union member states, residents in each of the member states, and aged 15 years and over. In these countries, the survey covers the national population of citizens of the respective nationalities and the population of citizens of the entire European Union member states that are resident in those countries and have a sufficient command of one of the respective national language(s) to answer the questionnaire. The basic sample design applied in all states is a multi-stage random one, totaling 27,524 respondents (see Table A1 in the Appendix A).

### 3.2. Measures

#### 3.2.1. Model Measurement Constructs (Distrust)

The survey measured vaccine trust (Cronbach’s Alpha = 0.77) using six items: “… how much trust you have in certain media and institutions…” Item 1. “The media”, Item 2. “Political parties”, Item 3. “Regional or local public authorities”, Item 4. “The national government”, Item 5. “The national parliament”, Item 6. “The European Union”. Respondents expressed their agreement with these statements on a two-item scale from 1 (“Totally agree”) to 2 (“Totally disagree”). As the scale was reversed, we named the resulting latent variable as DISTRUST.

#### 3.2.2. Mediator Variable (Populism)

The survey asked the interviewees three questions about political discontent (Cronbach’s alpha = 0.63). The first one (Item 7), measured on a four-point scale from 1 (“Totally agree”) to 4 (“Totally disagree”), was: “The interests of people like you are well taken into account by the political system in (OUR COUNTRY)”. The second one (Item 8), measured on a four-point scale from 1 (“Totally agree”) to 4 (“Totally disagree”), questioned “On the whole, are you very satisfied, fairly satisfied, not very satisfied, or not at all satisfied with the way democracy works in (OUR COUNTRY)?” The third one (Item 9), asked the participants “At the present time, would you say that, in general, things are going in the right direction or in the wrong direction, in (OUR COUNTRY?” We recoded this indicator in 1 (“Things are going in the right direction”), 2 (“Neither the one nor the other (SPONTANEOUS)), and 3 (“Things are going in the wrong direction”). As we commented in Section 2.2, political discontent and political populism are closely related concepts. Therefore, we use the first as a proxy for the latter.

#### 3.2.3. Outcome Variable (Vaccine Uptake)

The main outcome was the vaccine uptake reported by participants. It took the value 1 (“Yes”) if respondents answered affirmatively to either of the two survey questions “Have you had any vaccinations in the last five years?” and “Why have you not had any vaccination in the last five years? 1 You are still covered by vaccines you received earlier”. For the rest, it took value 0 “No”.

#### 3.2.4. Confounders

We included the following covariates to explore their effects on vaccine uptake: age (15–24 years, 8.2%; 25–39 years, 19.8%; 40–54 years, 24.5%; 55 years and older, 47.5%), gender (man, 45.3%; woman, 54.7%), age upon leaving full education (no full-time education, 0.7%; up to 15 years, 14%; 16–19 years, 43.3%; 20 years and older, 34.7%; still studying, 6%; missing values 1.3%), marital status (unmarried, 16%; (re-)married/single with a partner, 64.8%; divorced or separated, 8.2%; widowed, 10.4%; missing values, 0.6%), Occupation (self-employed, 6.9%; managers, 10.8%; other white collars, 12.5%; manual workers, 21%; house persons, 4.7%; unemployed, 5.2%; retired, 33%; students, 6%), residential setting (rural area or village, 33.7%; small or medium-sized town, 37.5%; large town, 28.7%), problems paying bills (most of the time, 8.3%; from time to time, 23.4%; almost never/never, 66.8%), social class (the working class of society, 26.4%; the lower-middle class of society, 15.3%; the middle class of society, 47%; the upper-middle class of society, 7%; the higher class of society, 0.6%), views on political matters/left–right positioning (left, 24.5%; center, 34.5%; right, 21.7%; missing values, 19.3%), use of online social networks (every day or almost every day, 14.4%; two or three times a week, 4.3%; about once a week, 1.9%; less often, 10.4%; never, 44.7%; missing values, 19.9%), children living at home (none, 76%; one, 11.8%; two, 9.1%; three, 2.2%; four or more, 0.8%).

All these data were obtained from the baseline survey.

### 3.3. Statistical Analysis

Structural equation modeling (SEM) examined the hypothesized mediating effects using Mplus software version 8.7 (Muthén & Muthén, Los Angeles, CA, USA). All the variables considered in the measurement model were treated as categorical. In accordance, we used the weighted least-squares estimator with mean and variance adjustments (WLSMV). We applied the probit link because the model’s dependent variable—vaccinated—was binary. The goodness of fit was assessed by computing the comparative fit index (CFI), Tucker–Lewis index (TLI), root mean square error of approximation (RMSEA), and standardized root mean residual (SRMR) [55]. The acceptable levels of the goodness-of-fit model parameters were CFI > 0.90, TLI > 0.90, RMSEA < 0.08, and SRMR < 0.08 [56]. Furthermore, to test the statistical significance of the mediating effects, we conducted bias-corrected bootstrap tests with 95% confidence intervals. We ran 20 different initial stage starts, and 10,000 bootstrap draws. The significance value was set at 0.05 in this study. The model also included all the potential confounders detailed previously.

## 4. Results

**Figure 2:**
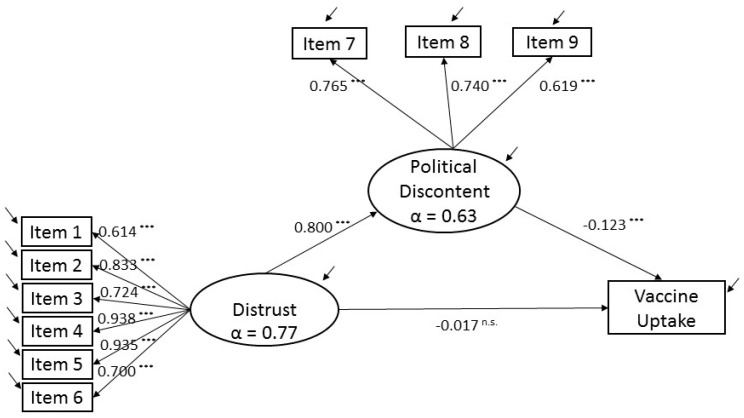
Structural Equation Model. Political populism mediation in the relationship between institutional distrust and vaccine uptake. Each latent variable has its associated Cronbach’s alpha (α). All path loads from latent variables to items are in standardized terms. *** represents *p*-values significant at the 1% level of significance. ^n.s.^ means no statistically significant results.

Figure 2 shows the final SEM model. This model was trimmed for the confounders that, in the first attempt, were not statistically significant in all the categories that each of them belonged to (gender, marital status, and residential setting). Fit statistics indicated that the SEM fitted the data well (χ^2^ = 5369.989, df = 312, CFI = 0.964, TLI = 0.959 SRMR = 0.054, RMSEA (90% CI) = 0.025 (0.024, 0.025)), and all standardized path coefficients were significant, except for the direct effect of DISTRUST on VACCINE. The model explained 9.1% of the variance of the vaccine uptake.

Mediation analysis was conducted to examine the mediation role of POPULISM on the relationship between DISTRUST and VACCINE UPTAKE (see Table 1). The total effect of DISTRUST on VACCINE UPTAKE (see Table 2) was statistically significant (standardized path coefficient, β = −0.115; *p* < 0.001; 95% CI, −0.133–−0.098). The indirect effect between DISTRUST and VACCINE UPTAKE was also statistically significant (standardized path coefficient, indirect effect = −0.0.098; 95% CI, −0.133–−0.063). Two effects that were also statistically significant composed this indirect effect: the effect of DISTRUST on POPULISM (standardized path coefficient, β = 0.800; *p* < 0.001; 95% CI, 0.790–0.811) and the effect of POPULISM on VACCINE UPTAKE (standardized path coefficient, β = −0.123; *p* < 0.001; 95% CI, −0.165–−0.079). Therefore, hypotheses 2 and 3 hold. Finally, the direct effect of DISTRUST on VACCINE uptake was not statistically significant (standardized path coefficient, direct effect = −0.017; *p* = 0.432; 95% CI, −0.060–0.025). Hence, hypothesis 1 holds, and we conclude that Populism completely mediates the association between Distrust and Vaccine Uptake.

Table 2 shows how the different sociodemographic variables considered in our study affected vaccine uptake. Because these variables were categorical, we needed to transform them into dummy variables to perform the probit analysis. To facilitate the interpretation of the coefficients, we converted the probit coefficients into logit ones, following Muthén and Muthén [57] (p. 43). This was done by applying the formula $logit\hat{\beta }=probit\hat{\beta }*\sqrt{\pi^{2}/3}$ [58] (p. 234). Finally, we obtained the odds ratio by exponentiating the logit coefficients (e^logit^).

Looking into the results depicted in Table 2, we see that odds ratios for vaccine uptake, adjusted for age, were lower for all the groups when compared to the youngest. Hence, participants between 25–39 years and 55 years and older had 13.8% (OR = 0.862, *p* < 0.001) lower odds than people between 15–24 years. Respondents between 40–54 had 16.4% lower odds than the reference group.

The second control variable, occupation, showed three categories that were not statistically significant: other white collars (OR = 1.011, *p* = 0.637), manual workers (OR = 0.998, *p* = 0.962), and house persons (OR = 0.977, *p* = 0.180). From the rest, there were three categories with higher odds (managers (OR = 1.071, *p* < 0.05), retired (OR = 1.065, *p* < 0.05), and students (OR = 0.083, *p* < 0.05)) and only the unemployed had lower odds for being vaccinated (OR = 0.940, *p* < 0.001).

Education was measured by answering the question at what age participants stopped full-time education. It was statistically significant for all the alternatives, except those consisting of people still studying (OR = 0.989, *p* = 0.511). The relationship between education and vaccine uptake was negative: higher education meant lower vaccination odds. Hence, when compared with respondents with no full-time education, participants that received education up to 15 years old had 4.1% higher odds to be jabbed (OR = 1.041, *p* < 0.05), those that stopped between 16 and 19 years old had 3.2% lower odds (OR = 0.968, *p* < 0.05), and participants with a higher education background had 16.6% lower odds to be vaccinated (OR = 0.844, *p* < 0.001).

Having children living at home represented higher odds to be vaccinated for one or two children, compared with families that had none (one (OR = 1.041, *p* < 0.05), two (OR = 1.033, *p* < 0.05)). However, the results for families with three or more children were not statistically significant (three (OR = 1.020, *p* = 0.172), four or more (OR = 0.993, *p* = 0.631)).

The economic situation of the interviewee also affected vaccine uptake. Those who declared problems with paying bills most of the time had 11.7% lower odds (0.883, *p* < 0.001). Furthermore, people that said that they had problems from time to time had 17.5% lower odds to be jabbed (OR = 0.825, *p* < 0.001) than people that said that they almost never/never had problems.

Social class was associated with vaccine uptake. Except for the higher social class that did not present statistically significant results (OR = 1.026, *p* = 0.084), we observe that the probability of being vaccinated was also higher as social class increased. Compared with the working class, the lower-middle class had 6.3% higher odds (OR = 1.063, *p* < 0.01), the middle class had 7.7% higher odds (OR = 1.077, *p* < 0.001), and the upper-middle class had 18.1% higher odds (OR = 1.181, *p* < 0.001) of being jabbed.

Political orientation was also related to vaccine uptake. The left-oriented participants were most likely to be vaccinated, with 11.9% higher odds than the center-oriented ones (OR = 1.119, *p* < 0.001). Results for right-oriented people were not statistically significant.

The use of online social networks did not shed any clear conclusion about its relationship with vaccine uptake. When compared to those who said they used online social networks every day or almost every day, three out of the five options available were not statistically significant (two or three times a week (OR = 0.029, *p* = 0.066), about once a week (OR = 1.002, *p* = 0.912), and two or three times a month (OR = 0.991, *p* = 0.572)). Participants who chose the less often (less than two or three times a week) option had 4.8% lower odds to be vaccinated than the reference group. The only clear insight we obtained was from comparing participants who never used online social networks with people who use them every day. The former had 10.5% greater odds of being vaccinated than the latter. Therefore, it depicted a clear difference between people who use or do not use online social networks. Still, there were no statistically significant differences among the different frequencies of use of online social networks, except for those who declared to use them less often than two or three times a week.

**Table 2:** Vaccine uptake confounders.

Variable	Categories	Probit	*p*	Logit	e^logit^
Age	15–24 years	Ref.	Ref.	Ref.	Ref.
	25–39 years	−0.082	0.000	−0.148	0.862
	40–54 years	−0.099	0.000	−0.179	0.836
	55 years and older	−0.082	0.000	−0.148	0.862
Occupation	Self-employed	Ref.	Ref.	Ref.	Ref.
	Managers	0.038	0.001	0.069	1.071
	Other white collars	0.006	0.637	0.011	1.011
	Manual workers	−0.001	0.962	−0.002	0.998
	House persons	−0.013	0.180	−0.024	0.977
	Unemployed	−0.034	0.000	−0.062	0.940
	Retired	0.035	0.036	0.063	1.065
	Students	0.044	0.001	0.080	1.083
Education	No full-time education	Ref.	Ref.	Ref.	Ref.
	Up to 15 years	0.039	0.000	0.071	1.073
	16–19	−0.018	0.045	−0.033	0.968
	20 years and older	−0.094	0.000	−0.170	0.844
	Still studying	−0.006	0.511	−0.011	0.989
Childs Living at Home	None	Ref.	Ref.	Ref.	Ref.
	One	0.022	0.007	0.040	1.041
	Two	0.018	0.037	0.033	1.033
	Three	0.011	0.172	0.020	1.020
	Four or more	−0.004	0.631	−0.007	0.993
Problems Paying Bills	Most of the time	−0.069	0.000	−0.125	0.883
	From time to time	−0.106	0.000	−0.192	0.825
	Almost never/never	Ref.	Ref.	Ref.	Ref.
Social Class	The working class	Ref.	Ref.	Ref.	Ref.
	The lower middle class	0.034	0.000	0.062	1.063
	The middle class	0.041	0.000	0.074	1.077
	The upper middle class	0.092	0.000	0.167	1.181
	The higher class	0.014	0.084	0.025	1.026
Political Left-Right	Left	0.062	0.000	0.112	1.119
	Center				
	Right	0.015	0.059	0.027	1.028
Use Online Social Network	Every day or almost every day	Ref.	Ref.	Ref.	Ref.
	Two or three times a week	0.016	0.066	0.029	1.029
	About once a week	0.001	0.912	0.002	1.002
	Two or three times a month	−0.005	0.572	−0.009	0.991
	Less often	−0.027	0.003	−0.049	0.952
	Never	0.055	0.000	0.100	1.105

Note: Dummy variables were created to perform the analysis. Ref. means the selected reference group. Logit coefficients were calculated from probit coefficients applying the formula $logit\hat{\beta }=probit\hat{\beta }*\sqrt{\pi^{2}/3}$. [58,59].

## 5. Discussion

This article explored the relationship between institutional distrust and vaccine uptake by recognizing the mediating role of political discontent (used as a proxy for measuring political populism). This research carried out SEM path analysis by using MPLUS 8.7. We found that institutional distrust was a significant predictor of vaccine uptake. Furthermore, the results also depicted that political populism fully mediated the relationship between institutional distrust and vaccine uptake. These outcomes corroborated the relationship observed by Kennedy [60]. Our research completes his work because we used data at the individual level, instead of macro data at the national level, for measuring populism. Additionally, we measured one of the main drivers of vaccine hesitancy [22], distrust of institutions, and its influence on vaccine uptake. Moreover, we went one step further, demonstrating that the effect of distrust on vaccine uptake was fully mediated by populism.

The consequences of our findings are clear: populist political parties could use vaccines as a battlefield because, when generating distrust in institutions, citizens with a higher level of political discontent had 11.5% lower odds of being vaccinated. It seems to be more than enough for reaching the destabilizing goals that populists pursue. Moreover, if the minimum herd immunity coverage is not achieved, new outbreaks will appear, reinforcing a vicious circle of distrust in elites [23,33]. These results are in line with several investigations that have studied the relationship between trust and vaccine uptake [25,26,61,62,63,64], while broadening and deepening the understanding of this link through the mediation role of political populism.

When analyzing the confounders, the results help predict whom populist political parties could target with their political marketing campaigns. The best profile for populists’ purposes was that of people older than 25 years old, unemployed, that stopped their full-time education at 20 years old and older (highly educated), with problems for paying bills, that declared to belong to the working class, politically oriented to the center, and that use online social networks. From this profile, the economic variables (unemployed, problems paying bills, and belonging to the working class) arose as the more important ones for explaining not being vaccinated, in odds terms [65]. In a few words, in Europe, the disenchanted from the global economy are the optimal target for populist political campaigns.

In Europe, traditionally, populists have been categorized as “radical right” or “extreme right” [66]. On the other hand, some other populist leaders have been considered economically left-wing, oriented mainly in Central and Eastern Europe [67]. Our results showed that politically center-oriented European citizens have greater odds of being attracted by distrusting populists’ proclaims to reject the vaccines. Hence, independently of the political orientation of the populist political party that tries to use distrust for campaigning, the main target will be the disenchanted and the politically center oriented.

The use of online social networks reduces the odds of being vaccinated compared to those who never use them. This result is congruent with several investigations [68,69] and shreds of evidence from the populist realpolitik [7]. Nevertheless, the frequency of use of these online social networks did not report any statistically significant difference. The communication media mix depends on the vaccine hesitancy segment that citizens belong to [70].

## 6. Conclusions

To reduce the adverse effects of these populist political strategies, it is necessary to reinforce citizens’ institutional trust. Public health has used, and should continue doing so in the future, health education, health promotion, and social marketing, as effective tools for influencing behavior, in the fight against several communicable and non-communicable diseases [71,72,73,74]. As the evidence shows, compulsory measures to vaccinate hesitant people have never been the answer to the lack of confidence in vaccines [70,75,76,77,78]. Evidence indicates that social marketing has considerable value in voluntarily fostering vaccine acceptance [72,73,79]. These actions, necessarily, have to be accompanied by an improvement in the living standards of these citizens, harmed by the globalization process [80,81]. It seems not to be a lack of trust in the health system, in particular, but rather in the political and economic institutions that the populists are taking advantage of for instilling doubt and trying to gain advantage from polarized debates about vaccines [48].

## 7. Limitations

This study should be evaluated with its limitations. The clearest one is using the Eurobarometer’s predefined items. Nevertheless, the benefit of the large-scale surveys, carried out by well-known public international organizations, is the high quality of the data obtained through a standardized sampling procedure. The authors intended to fill the gap that existed in the vaccine literature for testing the mediating role of political populism in the relationship between institutional distrust and vaccine uptake. For this purpose, Eurobarometer’s data fit perfectly.

Our results showed that the institutional distrust with the mediating role of political populism partially explained vaccination uptake. However, this implies that other variables also sway an individual’s decisions, since several circumstances finally influence vaccine uptake.

## Figures and Tables

**Table 1:** Direct, Indirect, and Total Effects of Distrust on Vaccine Uptake.

Paths	Unstandardized Path Coefficient, β Estimate (95% CI)	Standardized Path Coefficient, β Estimate (95% CI)	*p*
Direct Effect			
D → V	−0.029 (−0.102 0.043)	−0.017 (−0.060 0.025)	0.432
Indirect Effect			
D → P → V	−0.166 (-0.225 −0.107)	−0.098 (−0.133 −0.063)	<0.001
Total Effect			
D → V	−0.195 (−0.225 −0.165)	−0.115 (−0.133 −0.098)	<0.001

Abbreviations: CI, Confidence Interval; D, Distrust; P, Populism; V, Vaccine Uptake.

## Data Availability

Publicly available datasets were analyzed in this study. These data can be found through GESIS (University of Cologne, Germany) at https://www.gesis.org/en/eurobarometer-data-service/search-data-access/data-access (accessed on 16 October 2021).

## Appendix A

**Table A1:** Sample size by country, Total population older than 15 years (15+).

Country	Number of Interviews	Population 15+
Austria	1006	7,554,711
Belgium	1041	9,693,779
Bulgaria	1026	6,537,535
Croatia	1010	3,796,476
Czech Republic	1068	9,238,431
Denmark	1017	4,838,729
Estonia	1005	1,160,064
Finland	1000	4,747,810
France	1013	54,097,255
Germany	1507	70,160,634
Greece	1014	9,937,810
Hungary	1030	8,781,161
Ireland	1078	3,592,162
Italy	1021	52,334,536
Latvia	1012	1,707,082
Lithuania	1004	2,513,384
Luxemburg	512	457,127
Malta	497	364,171
Netherlands	1017	13,979,215
Poland	1011	33,444,171
Portugal	1013	8,480,126
Republic of Cyprus	505	741,308
Romania	1025	16,852,701
Slovakia	1020	4,586,024
Slovenia	1016	1,760,032
Spain	1014	39,445,245
Sweden	1021	7,998,763
United Kingdom	1021	52,651,777
TOTAL	27,524	431,452,219

Source: Eurobarometer 91.2. European Commission [54].
